# Down-film as a new non-frame porous material for sound absorption

**DOI:** 10.1038/s41598-024-62526-w

**Published:** 2024-05-21

**Authors:** Tingying Zhang, Jiyang Zhang, Hong Hou, Ying Xu, Kean Chen

**Affiliations:** School of Marine Science and Technology, Northwestern Polytechnical University, Xi’an, Shaanxi China

**Keywords:** Down, Film, Non-frame, Vibration, Sound absorption performance, Computational methods, Self-assembly

## Abstract

Down-polyethylene film material has been introduced for the first time as an excellent non-frame sound absorber, showing a distinctively outstanding performance. It contains down fiber adjacent to each other without firm connection in between, forming a structure of elastic fiber network. The unique structure has broadband response to sound wave, showing non-synchronous vibration in low and middle frequency and synchronous vibration in middle and high frequency. The broadband resonance in middle and high frequency allows the structure to achieve complete sound absorption in resonance frequency band. Moreover, down-polyethylene film material possesses forced vibration, corresponding sound absorption coefficient has been obtained based on vibration theory. The down-film sound absorption material has the characteristics of light weight, soft, environment-friendly, and has excellent broadband sound absorption performance.

## Introduction

Numerous sound absorption materials have been developed to alleviate the rapidly increasing noise pollution. The fundamental of sound absorption materials is to absorb sound via the attenuation of sound energy^1–4^. There are several conventional ways^5–7^ to obtain the attenuation of sound energy: the conversion to heat through the friction between material surface and air molecules, the consumption of sound energy by cavity resonance and film vibration, etc. In order to improve sound absorption performance, it has become a hotspot for decades to investigate new ways of sound energy conversion.

Porous materials display distinctive properties with high average sound absorption coefficients and broadband sound absorption, such as melamine foam^8–10^. Widely acceptable sound absorption mechanism of porous materials is the heat conversion from sound energy to friction between vibrated air molecules and material channel walls^11–13^, which is called friction sound absorption mechanism. Studies have been carried out on the optimization of thickness and porosity, in order to improve porous material sound absorption^14–16^. Research on the channel structure shows that increasing the complexity of the channel can improve the absorption performance^17–19^.

The structure of porous materials is mainly based on the frame structure, such as the foam materials and porous fiber materials^20,21^. This structure is whole and complete to ensure the structural stability of the materials. The channel in frame is the essential factor for sound absorption. More complex channel structures lead to more friction and therefore better sound absorption properties. In order to realize the complexity of channels, the thickness of porous materials has increased to improve the sound absorption properties^22^. Stable frame structures, such as rigid frame foam aluminum^23,24^, barely have secondary acoustic radiation because of the unlikeliness of structure vibration, resulting in a very efficient sound adsorption. Unstable frame structures, such as foam plastic with soft frame^25,26^, can readily vibrate and generate secondary acoustic radiation leading to a decrease in absorption performance. But, the vibrations of soft frame structure can still consume sound energy, so the sound absorption mechanism of the soft frame is relatively complex.

Apart from porous materials, cavity resonance materials also show excellent sound absorption properties. Based on the fundamental of Helmholtz resonator^27,28^, the conversion of cavity resonance is from sound energy to friction between sound waves and the wall of resonant cavities^29,30^. Microperforated plate is a distinctive cavity resonance structure, having wider band sound absorption than common resonance absorber^31^. Its micropores provide acoustic resistance while the resonance of air layer behind plate provides acoustic reactance. Thus, sound absorption under certain frequency can be achieved according to the theory of Dayou Ma. However, microperforated plate still has a relatively narrow sound absorption band for broadband noise^32^.

Film has been commonly used in sound transmission via secondary radiation^33,34^, such as paper cones in loudspeakers. Since its light weight, film structures can easily vibrate and transmit nearly all sound, therefore, barely absorb sound energy. The combination of film and cavity structure can be used as sound absorber to achieve resonance^35,36^. The film is to transmit sound waves to the cavity where the sound energy would be consumed via cavity resonance. Recent years, researchers have designed a composite structures of metal mass and films as acoustic metamaterial to achieve sound absorption at low frequency^37^. The sound energy on the film would be converted to vibration, of which the certain frequency would be consumed by the metal mass vibration. The absorbed sound frequency range depends on the weight of the metal mass^38,39^. Hence, it is very efficient to apply film during the process of sound conversion and transmission.

Bionics has been developing for decades since nature provides inspiration for humans. For instance, the invention of airplanes was derived from the imitation of birds. People have discovered the secret of owls’ silent flight, that is, feathers of their wings can divert the air flow and decrease fluid friction noise. Moreover, human’s ears have very interesting and complex structure, allowing people to hear sound between 20 and 20,000 Hz frequency^40^. The inner cochlea of vertebrate contains hair cell, of which the surface grows cilia bundles. When sound travels pass, the cilia would have mechanical movement, stimulating hair cell to have bioelectric response which would generate electrical signal^41,42^. The signal would be transmitted to brain and therefore sound can be heard. Since the cilia is super tiny and thin, even slight disturbance can cause its movement, humans can hear sound lower to 20 Hz. Thus, cilia can convert sound energy to mechanical energy, in other words, it shows the capability of absorbing sound energy. If there is a very light material in the nature, it is highly likely that the sound energy can be converted to mechanical energy. Interestingly, nature down fiber satisfies this criterion. From the microstructure of nature down fiber^43,44^, its trunk contains plenty of ultralight fiber with diameters of 2–15 µm and lengths of 0.5–3.5 cm. The component of down fiber is elastic protein, expanding automatically in cavity. Under the disturbance of force, down fiber shows similar movement as the cilia, providing the possibility of sound conversion. Therefore, down is a promising candidate to convert sound energy to mechanical energy to achieve sound absorption. However, it is unlikely to use down alone as a sound absorber due to its unfixability. If down can be put in a certain space to allow its movement, the friction between the fibers can consume sound energy and achieve sound absorption.

Down is an environmentally friendly material and research has been carried out about its sound absorption properties. Lightweight non-woven down fiber composite mats have been fabricated by hot pressing down and polymer. Compared with other natural materials, it shows a lower density and higher sound absorption coefficient at 250–800 Hz frequency under the condition of the same thickness^45–47^. So far, all relevant studies have applied mechanical pressing to make the frame porous fiber material. The sound would be converted to heat via surface friction. However, this structure does not allow fibers to extend and vibrate freely, so that its unique vibration properties are unlikely to be used.

In order to dig in the fundamental of sound absorption, plenty of model calculation methods have been developed for porous material structures due to its complexity. Two models are commonly acceptable, which are JCA semi-phenomenological model^48,49^ and empirical model. JCA semi-phenomenological model helps to investigate acoustic propagation characteristics by building up porous material viscous interactions among different physical properties^50,51^, including airflow resistance, porosity, thermal characteristic length, etc. This model applies to frame porous sound absorption materials with precise calculate, however, it shows some restrictions in practical use due to the difficulties of attaining parameters^52–54^. Empirical model requires plenty of experimental data to build sound absorption coefficient empirical formula, i.e., Delany-Bazley model^55^. This model provides the expression for the propagation constant and acoustic impedance of fiber materials based on lots of experimental results, showing that acoustic characteristics of porous materials are monotonically related to airflow resistance^56–58^. Empirical model is relatively simple and provides accurate calculations based on the sound absorption mechanism of friction dissipation. In general, all of the state-of-the-art porous frame models are based on friction conversion mechanism.

To date, studies of porous materials mainly focus on frame structures both in theory and practice. To the best of our knowledge, there is no research investigating non-frame structures. This paper has designed a down-film non-frame sound absorption structure for the first time, combining natural down ultralight structure and polyethylene film. The effect of various parameters on sound absorption performance of this material has been studied. From the perspective of impedance characteristic spectroscopy, theoretical model has been established to obtain sound absorption coefficient, which reveals the sound absorption mechanism of down-film non-frame porous material. The down-film sound absorption material obtained in this paper has the characteristics of light weight, soft, environment-friendly, easy to process and convenient use, and has excellent broadband sound absorption performance.

## Experiment

### Structure design of down-film material

Commercial down is the product of poultry growth, which can be naturally degraded and is an environmentally friendly material. Down is scattered velvet, which cannot be directly used as sound absorption material. In order to realize the down absorb the sound wave, polyethylene film is used to prepare a pocket, and the down is put into it. The film pocket can automatically expand due to the elasticity of down, which constitutes a down-film material.

#### Selection of film material

**Figure 1:**
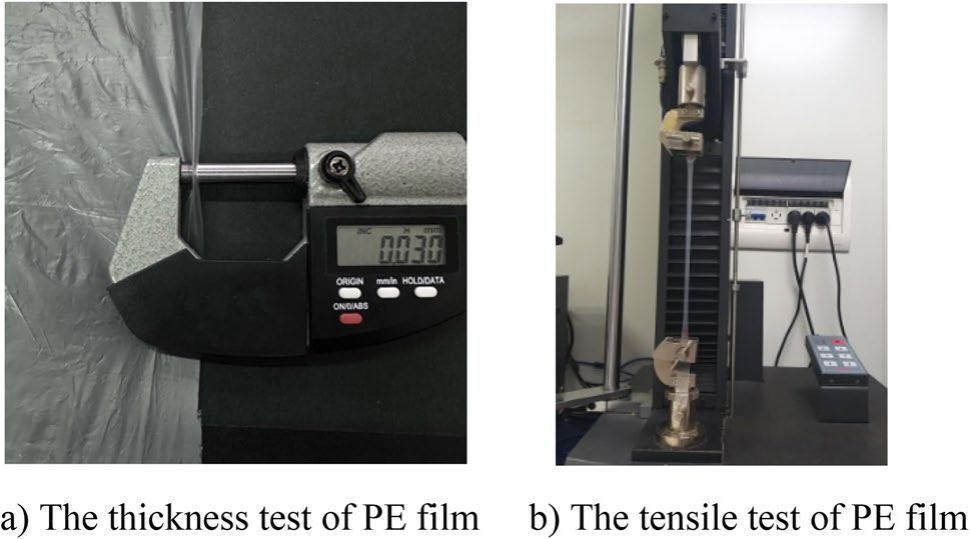
PE film.

Film structure can easily vibrate and transmit nearly all sound due to its light weight. Polyethylene (PE) film is selected in this experiment, which has the characteristics of light, soft and tensile. The tensile strength and elongation at break of PE film are tested, as shown in Fig. 1. The basic physical properties of PE film are shown in Table 1.

**Table 1:** The physical properties of PE film.

	Thickness/mm	Elongation to break/%		Tensile/N m^−1^	Density/g cm^−3^
		Vertical	Horizontal		
PE film	0.03	460	645	827	0.92

It can be seen from Table 1 that PE film has high tension and flexibility, and its thickness and density is low.

**Figure 2:**
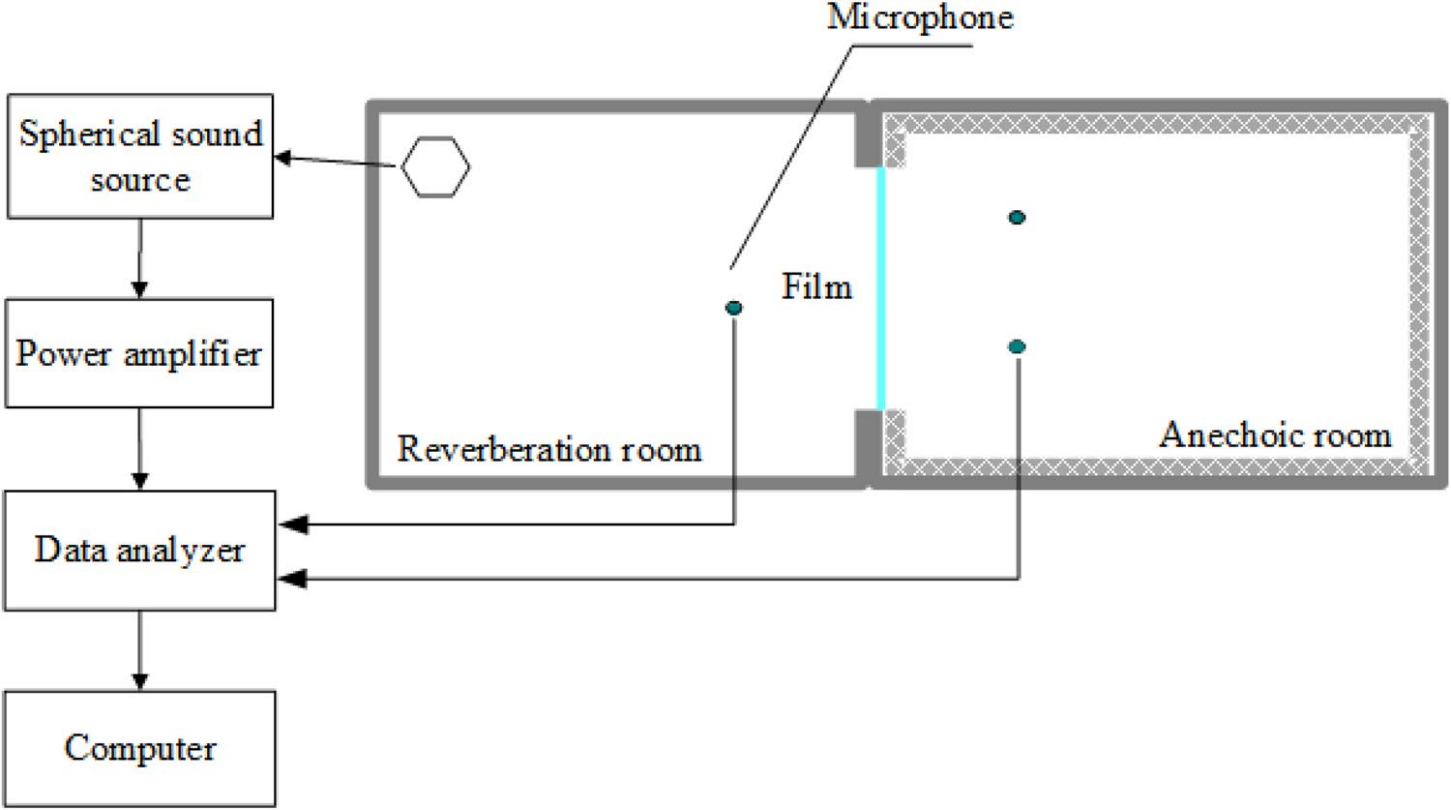
The test diagram for testing the sound transmission effect of PE film.

**Figure 3:**
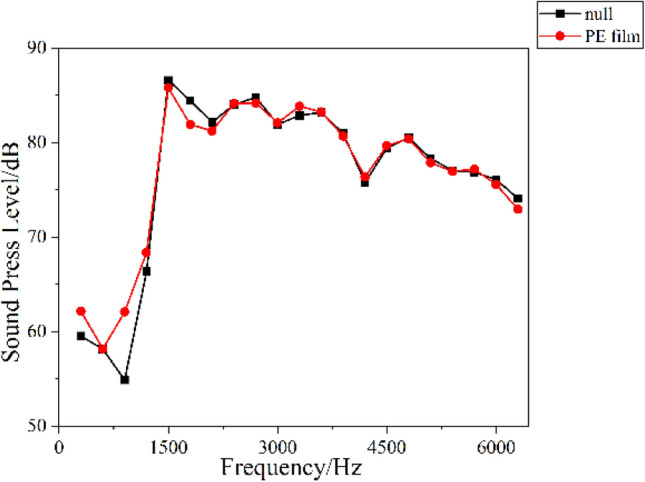
The test results of the sound transmission effect of PE film.

The film is installed in a test frame between the reverberation room and the anechoic room to test the transmission effect of PE film to sound waves. In the reverberation room, a spherical sound source is used for sound generation. Through the test frame, the response curves of the sound waves before and after the installation of the film can be obtained in the anechoic room. The test diagram is shown in Fig. 2, and the test results are shown in Fig. 3.

Figure 3 shows the test results of the sound transmission effect of 0.03 mm PE film. As shown in Fig. 3, the curves for the sound wave without and with the PE film remain nearly identical, indicating can easily transmit nearly all sound and barely attenuates sound waves. PE film can achieve the sound conversion and transmission via secondary radiation. During this transformation process, PE film can convert sound waves into vibration of the film, which will play an important role in the down-film material.

#### Nature down material

**Figure 4:**
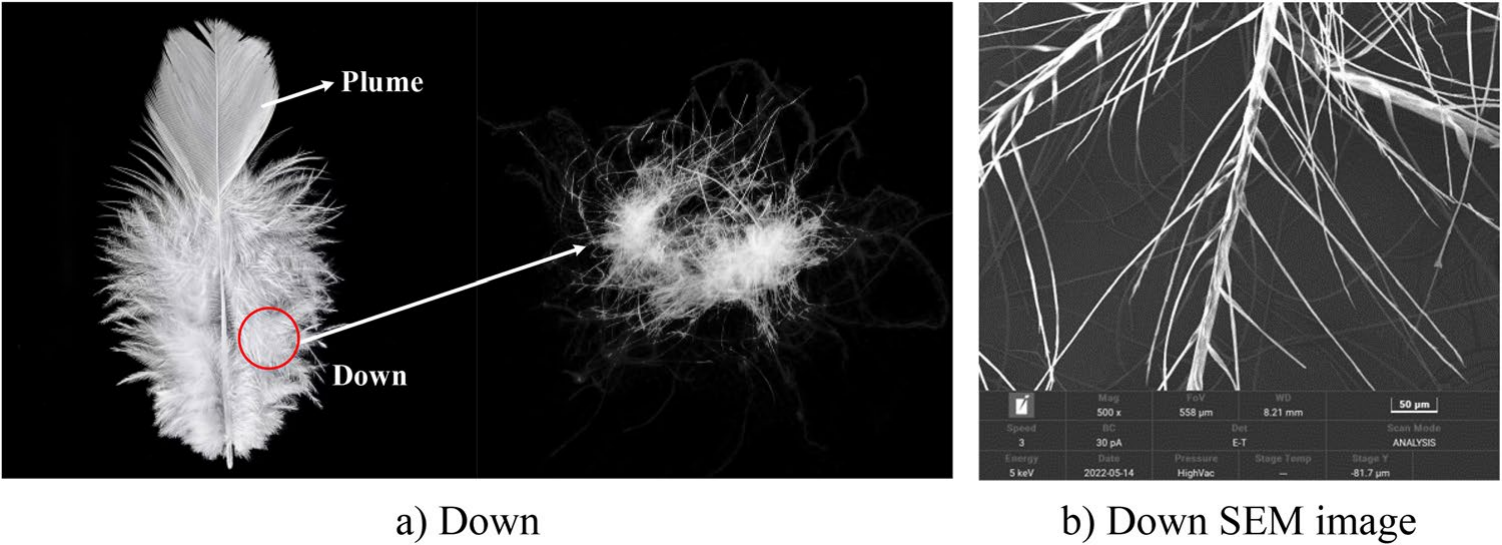
Nature down morphology.

Down is a part of poultry feathers that is ultra lightweight, elastic, and prone to movement when stimulated, as shown in Fig. 4a. The down component is an animal protein, and down produces a bundle structure during growth. This oriented bundle structure gives down fibers a certain strength and elasticity, so down automatic rebound. The microstructure of the down material is observed using scanning electron microscopy (SEM), and a 500-fold magnification of the down microstructure is shown in Fig. 4b.

It can be seen from Fig. 4 that nature down is a part of plume, and it is a form of velvet. From the microstructure of nature down fiber, its trunk contains plenty of ultralight fiber with diameters of 2–15 µm and lengths of 0.5–3.5 cm. Down is ultralight, soft and easy to move.

#### Preparation of down-film material

PE film and down material are selected, where PE film is used to make the external structure of the cavity, and the down material is inserted into it to form down-film material (DFM). The specific production process is to cut and paste the PE film into a cylinder with a base diameter of 29 mm, then calculate the required down quality based on the thickness and density of the sample (formula 1), fill it with down, and seal it.

**Figure 5:**
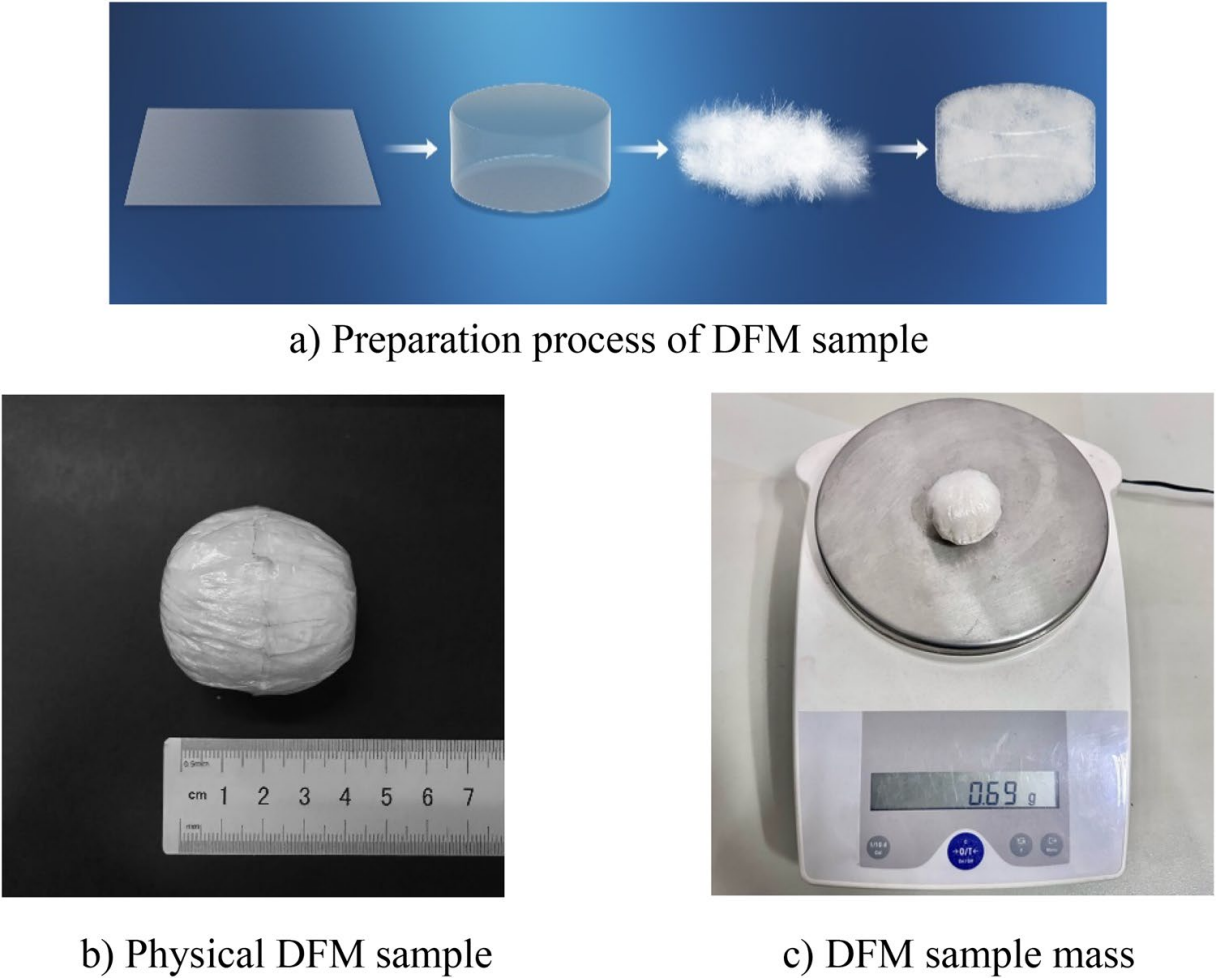
DFM sample.

The preparation process and physical samples are shown in Fig. 5.

1 $$ M_{D} = Sh_{D} \rho_{D} = 1.45^{2} \pi \times h_{D} \times \rho_{D} \times 10^{ - 3} $$

Among them, $S$ is the cross-sectional area of the down sample, $h_{D}$ is the thickness of down sample, $\rho_{D}$ is the down density.

### Sound absorption performance test

**Figure 6:**
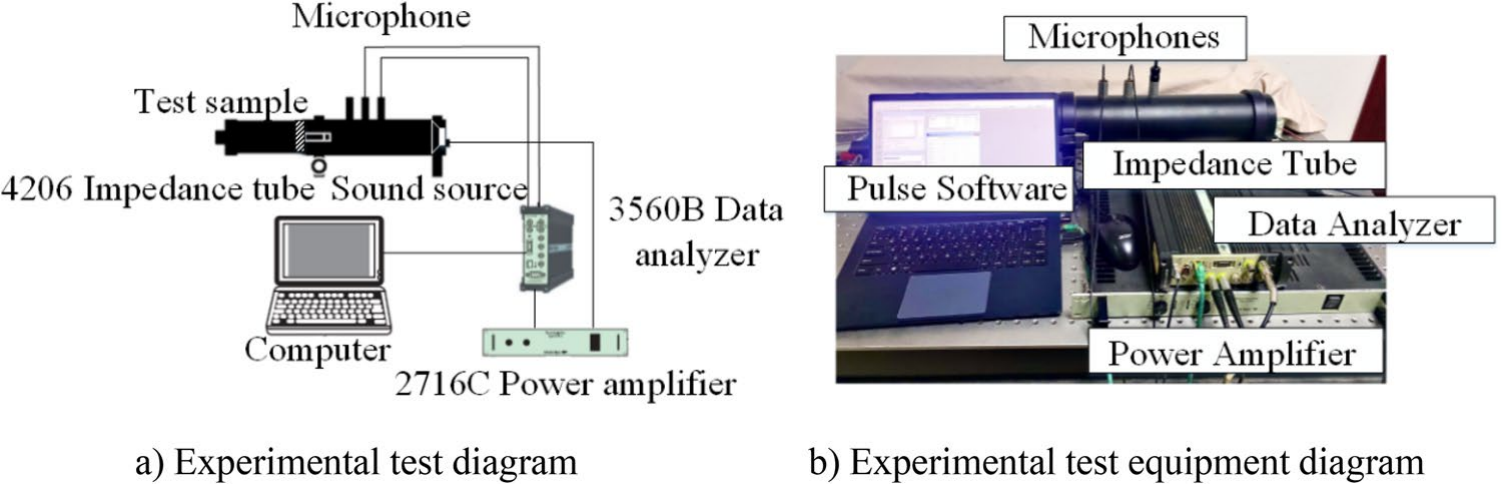
Experimental test.

The 4206 double microphone impedance tube of Danish B&K is used for the test, and the sound absorption coefficient of the sample is measured according to the transfer function method described in the Chinese national standard GB/T 18,696.2–2002. The test system consists of an impedance tube, two microphones, a power amplifier, a data analyzer and computer software, as shown in Fig. 6. During the experiment, the sample diameter is 29 mm, the test frequency is 500–6400 Hz, and the test interval of the sound absorption coefficient value is 8 Hz. The average sound absorption coefficient is the average value of sound absorption coefficient from 500 to 6400 Hz.

## Experimental results and discussion

### Effect of parameters on sound absorption performance of DFM

The main parameters affecting the sound absorption performance of DFM include the PE film thickness, sample thickness and density. The effects of various parameters on the sound absorption performance of DFM are as follows.

#### Effect of film thickness on sound absorption performance of DFM

**Figure 7:**
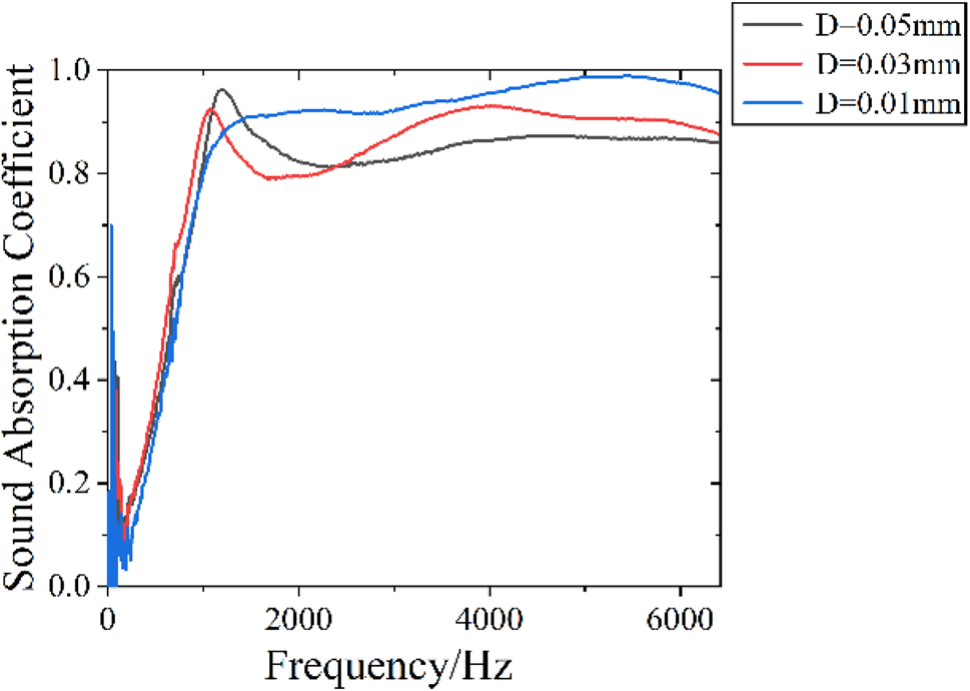
Sound absorption curve of DFM with different film thickness.

A sample thickness of 40 mm and a filling density of 0.02 g/cm^3^ are selected to prepare DFM with different film thicknesses of 0.05 mm, 0.03 mm, and 0.01 mm, and test the sound absorption coefficient. The experimental sample parameters and sound absorption performance results are shown in Table 2, and the sound absorption curve is shown in Fig. 7.

**Table 2:** Sound absorption performance results of DFM with different film thickness.

Film Thickness D/mm	Thickness $h$/mm	Density $\rho$/g cm^−3^	Average sound absorption coefficient $\alpha$	Frequency with sound absorption coefficient of 0.2 $f_{0.2}$/Hz
0.05	40	0.02	0.84	320
0.03	40	0.02	0.86	288
0.01	40	0.02	0.91	384

From Table 2 and Fig. 7, it can be seen that when the thickness of the DFM sample is 40 mm and the density is 0.02 g/cm^3^, the average sound absorption coefficient increases with the decrease of the film thickness. This is because the thinner film thickness, the better the sound transmission effect of the film, the better the sound absorption effect of the down fiber. When the film thickness is 0.01 mm, the average sound absorption coefficient reaches the maximum of 0.91. When the frequency is in 1352–6400 Hz, the sound absorption coefficient is greater than 0.9, showing almost perfect broadband sound absorption performance.

#### Effect of sample thickness on sound absorption performance of DFM

**Figure 8:**
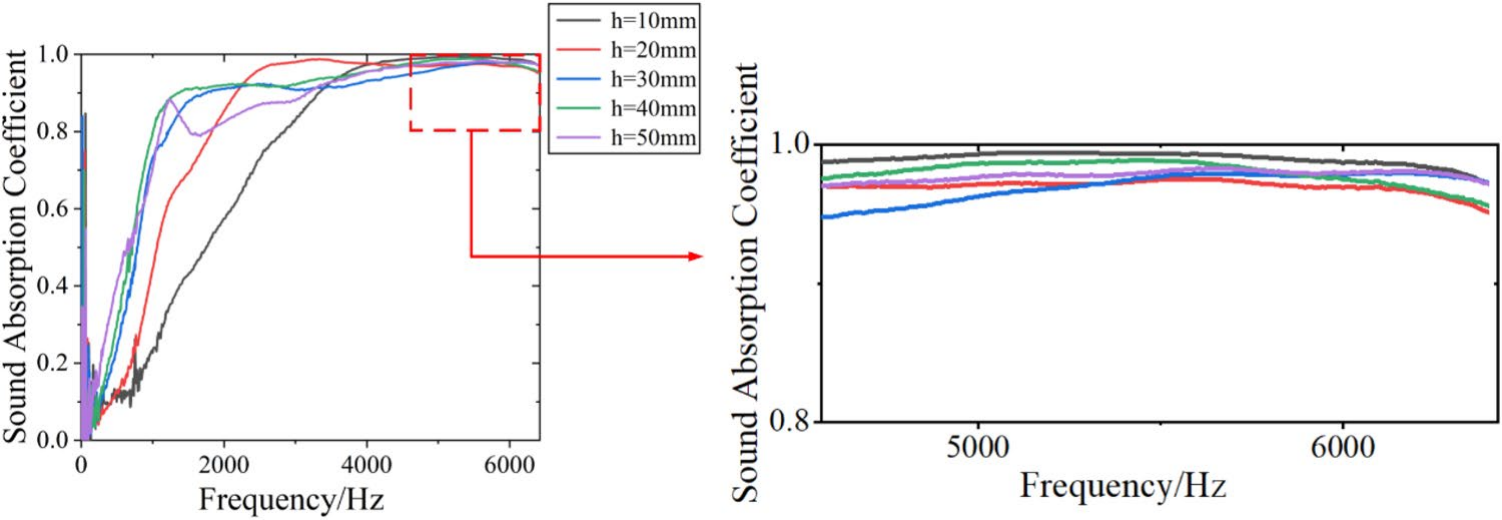
Sound absorption curve of DFM with different sample thickness.

When the film thickness and sample density are constant, the influence of sample thickness on the sound absorption performance of DFM can be obtained by changing the thickness of DFM. When the film thickness is 0.01 mm and the filling density is 0.02 g/cm^3^, the sound absorption coefficient of DFM with different sample thickness of 10 mm, 20 mm, 30 mm, 40 mm and 50 mm is tested. The experimental sample parameters and sound absorption performance results are shown in Table 3, and the sound absorption curve is shown in Fig. 8.

**Table 3:** Sound absorption performance results of DFM with different sample thickness.

Thickness $h$/mm	Film thickness D/mm	Density $\rho$/g cm^-3^	Average sound absorption coefficient $\alpha$	Frequency with sound absorption coefficient of 0.9 $f_{0.9}$/Hz	Frequency with sound absorption coefficient of 0.2 $f_{0.2}$/Hz
10	0.01	0.02	0.76	3320	912
20	0.01	0.02	0.86	2168	688
30	0.01	0.02	0.89	1744	440
40	0.01	0.02	0.91	1352	384
50	0.01	0.02	0.88	3160	272

It can be seen from Table 3 and Fig. 8 that with the DFM thickness increases, the sound absorption coefficient curve moves to low frequency, the average sound absorption coefficient is also increased, and the sound absorption performance in the medium and low frequency is improved. When the sample thickness is 40 mm, the average sound absorption coefficient reaches the maximum of 0.91, while when the thickness is 50 mm, the average sound absorption coefficient decreases, but the medium and low frequency is slightly better. The frequency corresponding to the absorption coefficient of 0.2 is the lowest frequency with sound absorption performance, and the lower it is, the better the low frequency sound absorption performance. It can be seen that as the thickness of DFM increases, $f_{0.2}$ moves to low frequency. When the thickness is 50 mm, $f_{0.2}$ can reach 272 Hz, so the low frequency sound absorption performance of DFM is better. When the sound absorption coefficient reaches 0.9, the lowest frequency is $f_{0.9}$. This is the lowest frequency for achieving excellent sound absorption performance. The lower it is, the wider the perfect sound absorption frequency band. When the thickness is 40 mm, $f_{0.9}$ is 1352 Hz, and the perfect sound absorption band is 1352–6400 Hz, with excellent sound absorption performance.

#### Effect of filling density on sound absorption performance of DFM

**Figure 9:**
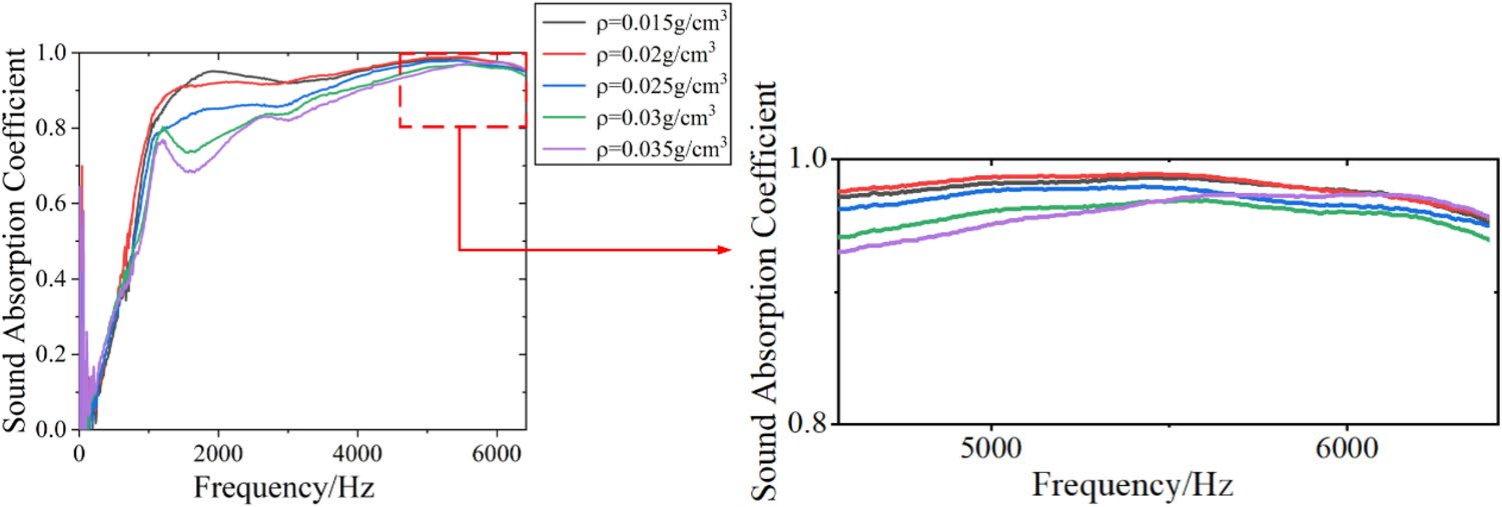
Sound absorption curve of DFM with different filling density.

When the film thickness is 0.01 mm and the sample thickness is 40 mm, the sound absorption coefficient of DFM with different filling density of 0.015 g/cm^3^, 0.02 g/cm^3^, 0.025 g/cm^3^, 0.03 g/cm^3^ and 0.035 g/cm^3^ is tested. The experimental sample parameters and sound absorption performance results are shown in Table 4, and the sound absorption curve is shown in Fig. 9.

**Table 4:** Sound absorption performance results of DFM with different filling density.

Density $\rho$/g cm^-3^	Film Thickness D/mm	Thickness $h$/mm	Average sound absorption coefficient $\alpha$	Frequency with sound absorption coefficient of 0.9 $f_{0.9}$/Hz	Frequency with sound absorption coefficient of 0.2 $f_{0.2}$/Hz
0.015	0.01	40	0.91	1432	416
0.02	0.01	40	0.91	1352	384
0.025	0.01	40	0.87	3448	368
0.03	0.01	40	0.84	3792	328
0.035	0.01	40	0.83	4024	316

It can be seen from Table 4 and Fig. 9 that with the DFM density increases, the average sound absorption coefficient and the sound absorption performance decreases. The higher the DFM density, the more down is contained in DFM. However, if the density of down is too high, the movement between fibers is limited, and the friction effect caused by the relative movement between down is reduced. Therefore, the sound absorption performance will be reduced. It can be seen that the density is 0.015 g/cm^3^ or 0.02 g/cm^3^, the average sound absorption coefficient of the sample is the same, both of which are 0.91, and the sound absorption performance reaches the best. The $f_{0.2}$ decreases with the increases of density. The higher the density, the slightly better the low frequency sound absorption performance. The $f_{0.9}$ increases with the increases of density. When the density is 0.02 g/cm^3^, $f_{0.9}$ reaches the minimum, so the perfect sound absorption band is the widest.

Taking into account all factors, the optimal parameters for DFM are: film thickness of 0.01 mm, sample thickness of 40 mm, filling density of 0.02 g/cm^3^. The sound absorption coefficient of DFM can reach 0.90 at 1352 Hz, and average sound absorption coefficient is 0.91. DFM has almost perfect broadband sound absorption performance.

## Sound absorption mechanism and theoretical calculation of DFM

### Impedance spectrum analysis of typical porous material and DFM

The sound absorption coefficient and sound absorption curve are usually used to evaluate the sound absorption material. A high average sound absorption coefficient indicates an excellent dissipation effect on sound waves. And from the sound absorption curve, the relationship between the sound absorption coefficient and frequency can be seen, which can evaluate the sound absorption performance in different frequency bands. But these parameters cannot obtain the real dissipation mechanism of material. While testing the sound absorption coefficient, the acoustic impedance spectrum of material can be measured. The impedance spectrum can observe the laws of material acoustic resistance and acoustic reactance, thus revealing the law of material acoustic attenuation. The acoustic impedance spectrum of materials with different frame structure are different. Acoustic resistance is the real part of acoustic impedance, and acoustic reactance is the imaginary part of acoustic impedance.

Frame structure refers to the overall framework structure composed of basic materials. Porous glass is a frame porous structure prepared from glass as the basic material. Since glass is a rigid material, the porous structure formed is called the rigid frame structure. The rigid frame will not vibration under the action of sound waves and will not occur secondary acoustic radiation^59^. Soft frame structure means that the basic material of the frame is soft, for example, melamine foam is a soft frame structure. The soft frame will vibrate under the action of sound waves, which will occur secondary acoustic radiation.

DFM is composed of down fibers. Due to the softness of down fibers and their interdependence without any fixed connections, DFM is a non-frame structure.

**Figure 10:**
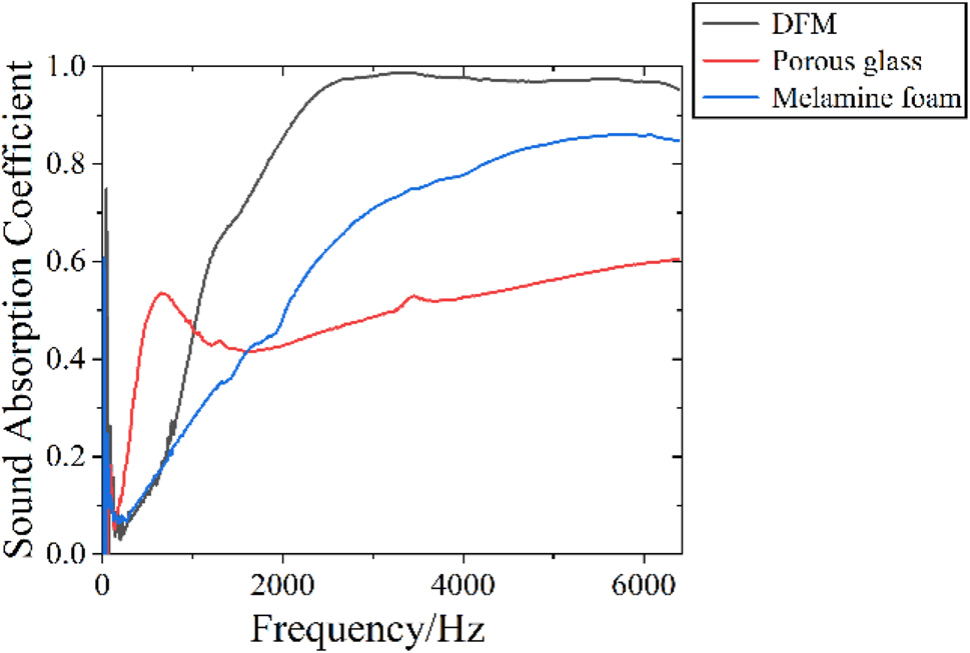
Sound absorption curve of materials.

**Figure 11:**
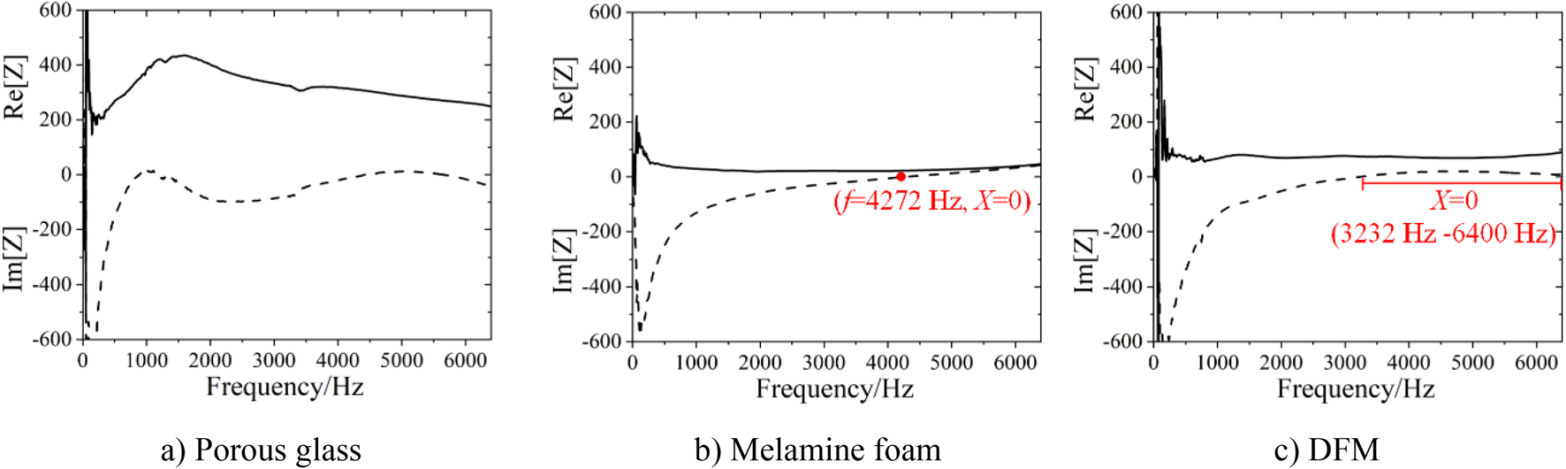
Acoustic impedance spectrum of materials.

Porous glass, melamine foam and DFM are selected for sound absorption coefficient measurement, in order to reveal the different sound absorption mechanism between general frame porous sound absorption materials and DFM. The parameters of these materials are shown in Table 5. The sound absorption curve and acoustic impedance spectrum are in Figs. 10 and 11.

**Table 5:** Parameter and sound absorption coefficient of materials.

Materials	Frame structure	Thickness $h$/mm	Density $\rho$/g cm^−3^	Average sound absorption coefficient $\alpha$	Frequency with sound absorption coefficient of 0.2 $f_{0.2}$/Hz
Porous glass	Rigid frame	20	0.672	0.51	280
Melamine foam	Soft frame	20	0.009	0.66	736
DFM	Non-frame	20	0.02	0.86	696

It can be seen from Table 5 and Fig. 10 that when the thickness of the three materials is 20 mm, the average sound absorption coefficient of DFM is the largest, 0.86. The average sound absorption coefficients of melamine foam and porous glass are 0.66 and 0.51 respectively. All three materials exhibit broadband sound absorption performance. The sound absorption performance of porous glass at medium and low frequencies is better than that of DFM and melamine foam.

It can be seen from Fig. 11 that the acoustic impedance spectrum of these materials is significantly different. From the theoretical calculation formula of sound absorption coefficient (formula 2), it can be seen that when the acoustic resistance exists, the maximum sound absorption coefficient can be obtained if the acoustic reactance is 0.

2 $$ \alpha = 1 - \left| {\frac{{Z - \frac{{\rho_{0} c_{0} }}{S}}}{{Z + \frac{{\rho_{0} c_{0} }}{S}}}} \right|^{2} = \frac{{4RS\rho_{0} c_{0} }}{{\left( {RS + \rho_{0} c_{0} } \right)^{2} + X^{2} S^{2} }} $$

Among them, $Z$ is the acoustic impedance of the structure, $R$ is the acoustic resistance, $X$ is the acoustic reactance. $S$ is the cross-sectional area of the structure, $\rho_{0} c_{0}$ is the air impedance, which is $415\,{\text{Pa}} \cdot {\text{s}}/{\text{m}}$.

Figure 11a shows the acoustic impedance spectrum of porous glass, where the sound resistance varies greatly with frequency, and the sound reactance also varies greatly with frequency. Porous glass is rigid frame structure, and the structure cannot vibrate. The sound absorption mechanism of porous glass is the heat conversion from sound energy to friction between vibrated air molecules and material channel walls, which is called friction sound absorption mechanism. The acoustic impedance varies complexly with frequency, and the acoustic resistance value is relatively large, with an average acoustic resistance value of about 322.35, which is caused by the complex structure of the rigid frame and the channels. Therefore, the viscous resistance of channel wall should be considered mainly in the sound absorption coefficient calculation of the rigid frame porous materials.

Figure 11b shows the impedance spectrum of melamine foam, where the acoustic resistance is almost a constant, about 29.35. It can be seen that the sound resistance in the rigid frame structure is large, while the sound resistance in the soft frame structure is small and independent of frequency. This means that in the melamine foam, the sound resistance caused by friction accounts for a small proportion, and its sound resistance is mainly caused by the vibration damping of the structure itself. The acoustic reactance gradually increases with frequency, and the acoustic reactance is 0 when frequency is 4272 Hz. Melamine foam is soft frame structure, which will vibrate under the excitation of sound waves, and its acoustic reactance varies with frequency.

Figure 11c shows the impedance spectrum of DFM, where the acoustic resistance is a constant, about 73.96. The acoustic resistance will not vary with frequency, indicating that the acoustic resistance of DFM is caused by vibration rather than friction between air molecules and channel walls. The acoustic reactance varies with frequency below 3232 Hz, but is almost 0 in the range of 3232–6400 Hz. At the same time, it can be seen from the sound absorption coefficient curve that the average sound absorption coefficient at 3232–6400 Hz is 0.97, almost completely absorbed. For a vibration system, the acoustic reactance of 0 indicates that the system has undergone resonance. Therefore, DFM exhibits broadband resonance characteristics in the range of 3232–6400 Hz.

Comparing the impedance spectrum of porous glass, melamine foam and DFM, it can be seen that the material with rigid frame has more complex acoustic impedance spectrum and maximum acoustic resistance. This is because the sound wave attenuation of rigid frame materials is a friction dissipation mechanism, in which the complex pore structure and the viscous resistance determine the impedance characteristics. The acoustic resistance of melamine foam is very small and independent of frequency, which is determined by the vibration damping inside the material. The acoustic reactance spectrum of melamine foam varies with frequency, which indicates that the melamine foam is a frame vibration and has different responses to different frequencies. The acoustic resistance spectrum of DFM is similar to that of melamine foam, in which the acoustic resistance is a very small constant and independent of frequency. Different from melamine foam, the acoustic reactance spectrum of DFM has a resonance frequency range. In this resonance frequency range, the acoustic reactance is 0, and the sound absorption coefficient reaches the maximum value, showing the perfect sound absorption performance. This is related to the structure of DFM.

**Figure 12:**
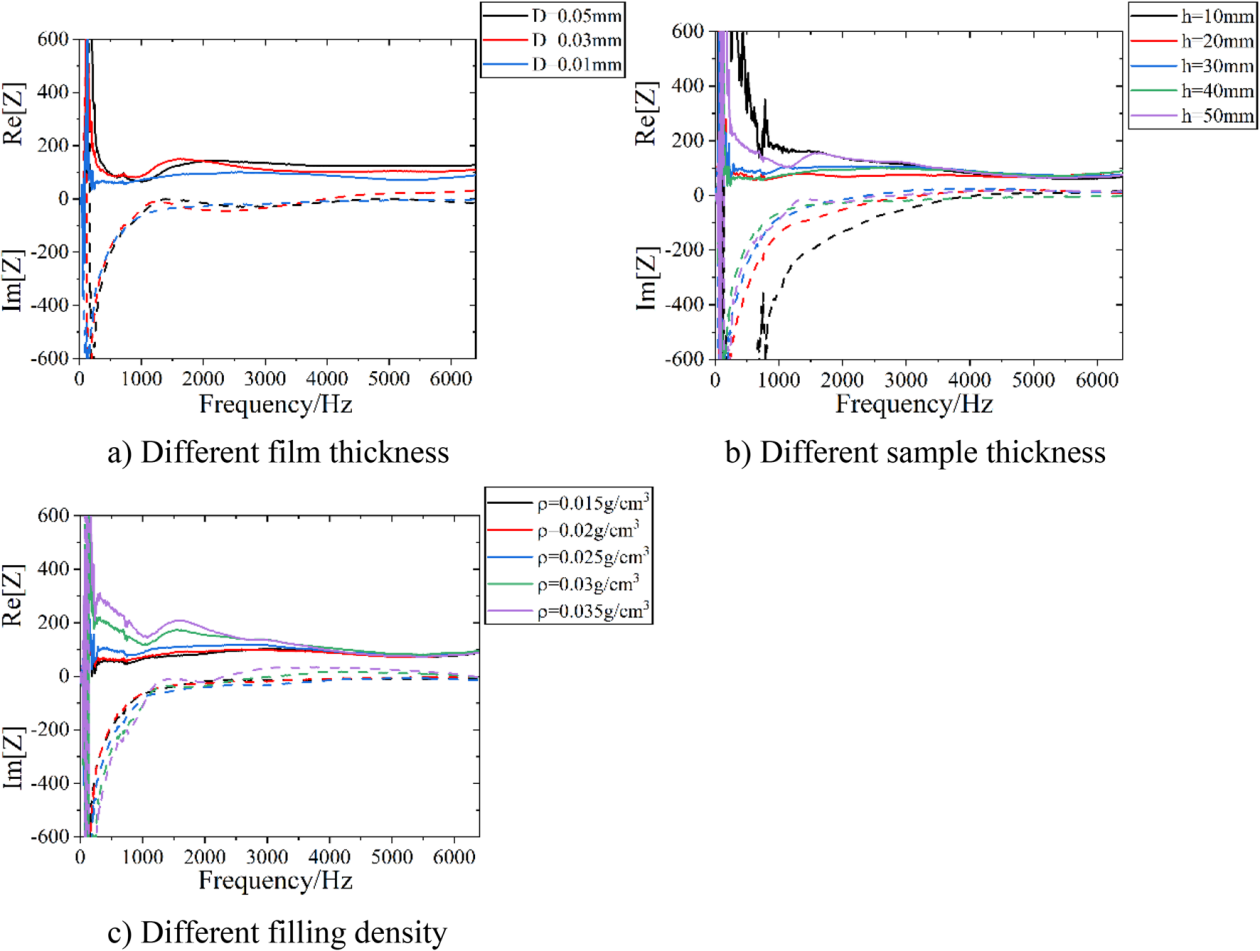
Acoustic impedance spectrums of DFM with different parameters.

For DFM with different parameters, the resonance frequency range varies. The acoustic impedance spectrums of DFM with different parameters are shown in Fig. 12.

It can be seen that the impedance spectrum of DFM with different thickness, density and PE film thickness has the same rule, and the resonance frequency range is different. The DFM with film thickness of 0.01 mm, thickness of 40 mm and density of 0.035 g/cm^3^ has the widest resonance frequency range, which is 2328–6400 Hz.

### Sound absorption mechanism of DFM

From the acoustic impedance spectrum of DFM, it can be seen that the acoustic reactance is 0 in a certain frequency range, and the absorption coefficient reaches its maximum in this frequency range. This is a resonance absorption characteristic, that is, the sound energy consumption is the highest at the resonance frequency. Due to the fact that the acoustic reactance is 0 over a wide frequency range, resonance occurs at all frequency points, which is the characteristic of broadband resonance.

From the structure of DFM, it contains down fiber adjacent to each other without firm connection in between, like an elastic fiber network composed of many independent micro-springs. This structure is complex, and each micro-spring can respond to different frequencies, resulting in non-synchronous vibration and synchronous vibration. Synchronous vibration occurs in the medium to high frequency, which generates resonance, forming the broadband resonance sound absorption.

The sound absorption mechanism of DFM is that sound waves are transmitted to down fibers through film vibration, causing non-synchronous vibration in low and middle frequency and synchronous vibration in middle and high frequency, resulting in broadband resonance. Vibration causes friction between fibers, consuming sound energy.

### DFM equivalent theory

**Figure 13:**
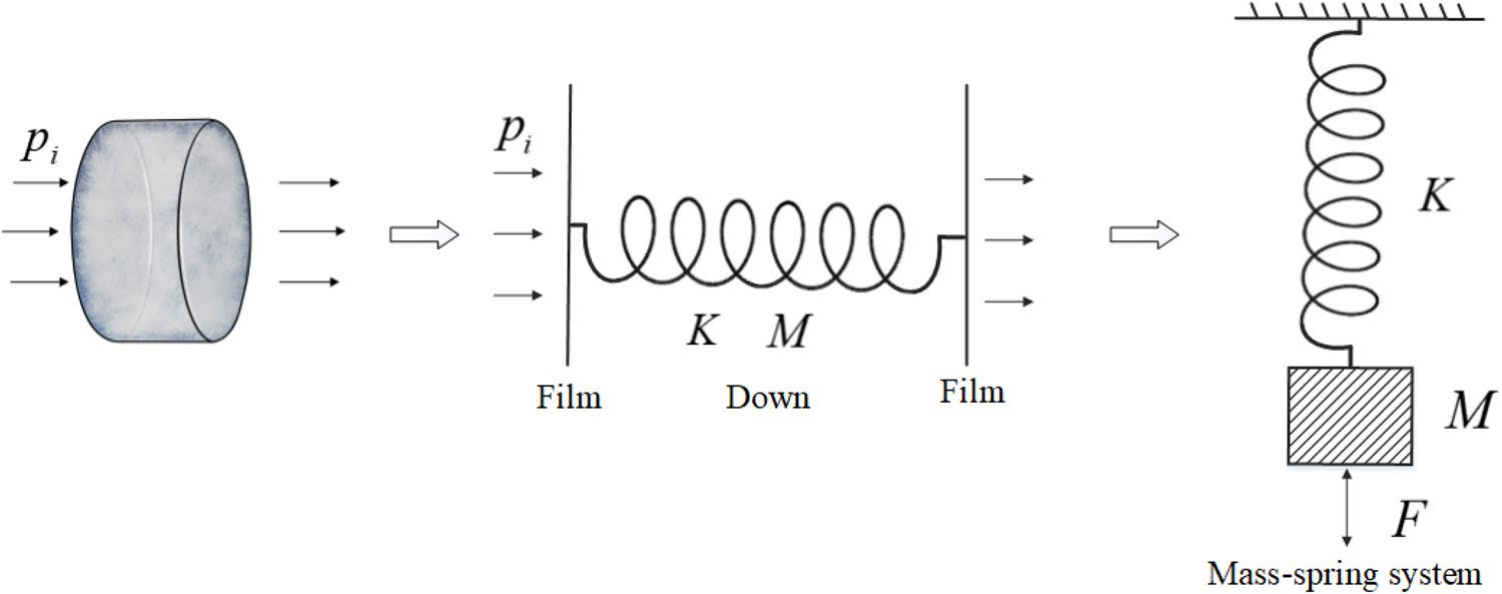
Down-film vibration system.

From the sound absorption mechanism analysis of DFM, it can be seen that DFM has typical forced vibration characteristics. DFM can be equivalent to a mass-spring system from a macro perspective, as shown in Fig. 13. $K$ is the elastic coefficient of the spring, $M$ is the mass of the system, $p_{i}$ is the incident sound wave.

$p_{i}$ acts on the mass-spring system, and the system occurs forced vibration. The vibration equation is:

3 $$ M\frac{{d^{2} \xi }}{{dt^{2} }} + R\frac{d\xi }{{dt}} + K\xi = p_{i} e^{j\omega t} $$

where $\xi$ is the displacement of the mass block in the mass-spring system leaving the balance position during vibration.

The forced vibration equation is a second-order non-homogeneous ordinary differential equation, and its solution should be expressed as the sum of a particular solution of this equation and the general solution of the corresponding homogeneous equation. Let the particular solution be:

4 $$ \xi_{1} = \xi_{F} e^{j\omega t} $$

where $\xi_{F}$ is the undetermined constant. By substituting this formula into formula (3), we can get:

5 $$ \xi_{F} \left( { - M\omega^{2} + R\omega j + K} \right) = p_{i} $$

6 $$ \xi_{F} = \frac{{ - jp_{i} }}{\omega Z} = \frac{{p_{i} }}{\omega \left| Z \right|}e^{{ - j\left( {\theta_{0} + \frac{2}{\pi }} \right)}} $$

When the sound wave is incident on the DFM surface, the DFM vibration system will occur forced vibration, and there is force impedance $Z = R + jX$. Among them, $R = \omega_{0} M$ is the force resistance, it can be equivalent to acoustic resistance, which comes from the vibration, swing and friction between down fibers caused by film vibration. $X = \omega M - \frac{K}{\omega }$ is force reactance, it can be equivalent to acoustic reactance, which comes from the vibration of film and down fibers. $\theta_{0} = \arctan \frac{X}{R}$ is its argument, $\omega$ is angular frequency of sound wave, $\omega = 2\pi f$.

In this vibration system, the parameters of DFM to be determined are: system mass $M$, elastic coefficient $K$ and system acoustic resistance $R$.

1. System mass $M$ is composed of film mass and down mass, namely:
  7 $$ M = M_{1} + M_{2} $$
  where the film thickness is 0.01 mm, $M_{1}$ is the film mass:
  8 $$ M_{1} = S_{F} h_{F} \rho_{F} = \left( {4.5\pi + 3\pi \times h_{D} } \right) \times 0.001 \times 0.92 \times 10^{ - 3} $$
  $M_{2}$ is the down mass:
  9 $$ M_{2} = Sh_{D} \rho_{D} = 1.45^{2} \pi \times h_{D} \times \rho_{D} \times 10^{ - 3} $$
  Among them, $S_{F}$ is the surface area of film, $h_{F}$ is the film thickness, $\rho_{F}$ is the film density, $S$ is the cross-sectional area of the down sample, $h_{D}$ is the thickness of down sample, $\rho_{D}$ is the down density.
2. Elastic coefficient $$K$$:
  10 $$ K = a \times M $$
  where $a$ is the correction factor:
  11 $$ a = \left( {6.42 \times 10^{4} + 3.22 \times 10^{7} \times \rho_{D} } \right) \times h_{D}^{ - 0.4} $$
3. Acoustic resistance $$R$$:
  12 $$ R = 40.71 + 3068 \times \rho_{D} $$
  when $M$,$K$ and $R$ of DFM system are determined, the vibration displacement characteristics of DFM can be obtained.

### Sound absorption coefficient theoretical calculation of DFM

In the research of sound absorption materials, the sound absorption performance of materials can be estimated by calculating the sound absorption coefficient.

For the sound absorption coefficient calculation of DFM, the acoustic resistance can be obtained firstly based on the vibration theory of DFM, and the value is as shown in formula (12). Secondly, the value of the acoustic reactance is divided into two parts in order to calculate accurately, one is the calculation of the non-resonance frequency range, and the other is the value of the resonance frequency range, namely:

13 $$ \begin{aligned} & {\text{Non - resonance}}\,{\text{frequency}}\,{\text{range:}}\,X = b \times M \times 2\pi f - \frac{c \times K}{{2\pi f}} \\ & {\text{Resonance}}\,{\text{frequency}}\,{\text{range:}}\,X = 0 \\ \end{aligned} $$

where $b$ and $c$ are correction factor:

14 $$ \begin{aligned} & b = 5.9 \\ & M \le 0.52 \times 10^{ - 3} kg,\quad c = - 300 \times \ln \left( {M \times 10^{3} - 0.13} \right) \\ & M > 0.52 \times 10^{ - 3} kg,\quad c = 150 \\ \end{aligned} $$

Finally, the sound absorption coefficient of DFM is calculated by formula (2).

### Verification of the sound absorption coefficient calculation formula of DFM

**Figure 14:**
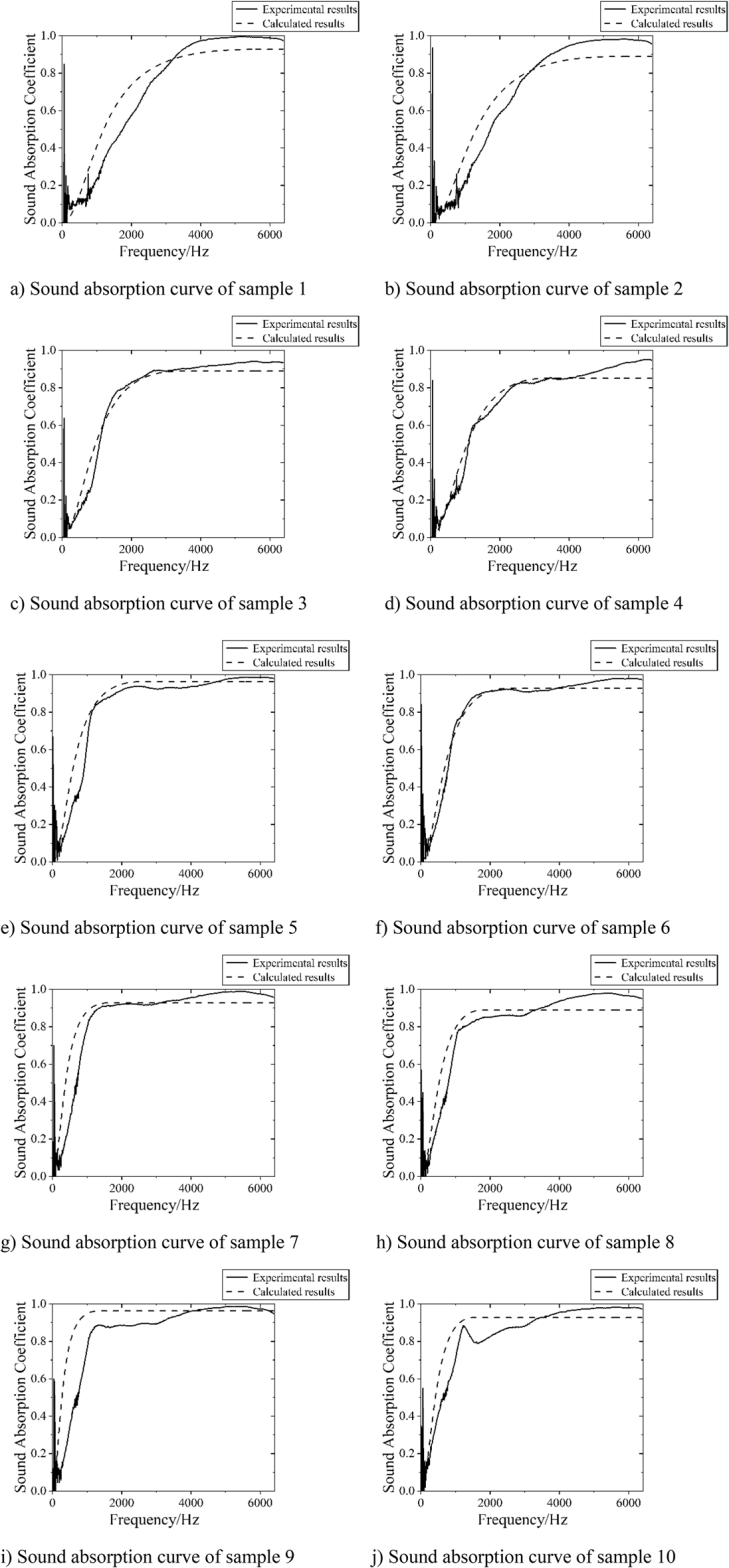
Comparison of DFM sound absorption curve calculation and experimental results.

The formula is used to calculate the sound absorption coefficient of the sample used in the experiment. The comparison of the sample parameters, calculation results and experimental measurement results is shown in Table 6, and the theoretical curve and experimental measurement curve are shown in Fig. 14.

**Table 6:** Comparison of calculation and experimental measurement results.

Sample	Thickness/mm	Density/g cm^−3^	Average sound absorption coefficient (500-6400 Hz)	
			Calculation	Experimental measurement
1	10	0.02	0.79	0.76
2	10	0.025	0.75	0.75
3	20	0.025	0.81	0.82
4	20	0.03	0.77	0.77
5	30	0.015	0.92	0.89
6	30	0.02	0.88	0.89
7	40	0.02	0.91	0.91
8	40	0.025	0.87	0.87
9	50	0.015	0.94	0.90
10	50	0.02	0.91	0.88

It can be seen from Table 6 that the average sound absorption coefficient calculated theoretically is basically the same as the experimental measurement results, and the maximum difference between the theoretical and experimental values is 0.04. It can be seen from Fig. 14 that the shape of the absorption curve calculated theoretically is consistent with that measured experimentally, and the calculation results are accurate. However, it can be seen that there are certain differences between the theoretically calculated and experimentally measured sound absorption curves at low frequencies. In theoretical calculations, it is believed that the film completely transmits sound to the down material. In the experiment, at low frequencies, the film is easier to vibrate, and the secondary sound radiation caused by the vibration is also larger, generating new sounds, resulting in an increase in the sound pressure level at low frequencies. These can be seen from the sound transmission effect curve in Fig. 3. Therefore, there will be a certain error between the experimental test results and the theoretical calculation values at low frequencies. Generally speaking, the sound absorption coefficient of DFM is calculated based on vibration theory, and very ideal calculation results are obtained.

## Conclusion

The down used in this paper is a naturally growing material of poultry, which is environmentally friendly. The sound absorption coefficient of non-frame structure DFM made of down and polyethylene film is related to the thickness of PE film, the filling density and thickness of sample. The thinner the thickness of PE film, the better the sound absorption performance. The density and thickness of DFM have the best range. In this study, DFM with an average sound absorption coefficient of 0.91 is obtained, which has almost excellent sound absorption performance.

From the structure of DFM, it contains down fiber adjacent to each other without firm connection in between, like an elastic fiber network composed of many independent micro-springs. This structure is complex, and each micro-spring can respond to different frequencies, showing non-synchronous vibration in low and middle frequency and synchronous vibration in middle and high frequency. The broadband resonance in middle and high frequency allows the structure to achieve almost complete sound absorption in resonance frequency band.

The sound absorption mechanism of DFM is that sound waves are transmitted to down fibers through film vibration, causing non-synchronous vibration in low and middle frequency and synchronous vibration in middle and high frequency, resulting in broadband resonance. Vibration causes friction between fibers, consuming sound energy.

DFM has elastic vibration characteristics, and its motion follows forced vibration. The forced vibration equation can be used to perfectly describe the vibration characteristics of DFM, in which three parameters are determined as system mass, acoustic resistance and elastic coefficient. According to the vibration theory, the sound absorption coefficient of DFM is calculated, and the ideal calculation results are obtained.

The down-film sound absorption material obtained in this paper has the characteristics of light weight, soft, environment-friendly, easy to process and convenient use, and has excellent broadband sound absorption performance.

## Data Availability

Data is provided within the manuscript and the original data can be provided from Tingying Zhang (tingying0902@mail.nwpu.edu.cn) upon request.
